# Supplementary material for: Ethological principles predict the neuropeptides co-opted to influence parenting
Source: Nat Commun. 2017 Feb 1;8:14225. doi: 10.1038/ncomms14225 (PMC5296637; doi:10.1038/ncomms14225)
Supplement: Supplementary Information — Supplementary Table 1 [file ncomms14225-s1.pdf]

Supplementary Table 1. Individual peptides with observed modifications and their associated neuropeptides.

Neuropeptide gene	Peptide	Possible modifications observed	Test of difference in neuropeptide abundances
Apis-ITG-like (ITG)	AGEKRLTGLAAFKRPMH	N terminus acetylation; Oxidation; C terminus amidation	$F_{2,9} = 2.826$, $P = 0.11$
	ALLAICLLGRQTEAWGGL	N terminus acetylation	
	ANMGYG	N terminus acetylation	
	LTGLAAFKRPM	Oxidation	
	TCLFAYGRRVGELCRRDSDCESGLVC	C terminus amidation	
	VCTESEQTSSSRICR		
Apis-NVP-like (NVP)	APVNAESHGESRPT		$F_{2,9} = 4.210$, $P = 0.05$
	APVNAESHGESRPTA		
	APVNAESHGESRPTAV	C terminus amidation	
	FAALALALPASVVEDVKSSDIKNSKVKRAP	C terminus amidation	
	FLNGPTRNNYYTLSELLGAAQQEQNVPLYQRYVL	C terminus amidation	
	LPASVVEDVKSSD	C terminus amidation	
	LPASVVEDVKSSDIKN		
	LPASVVEDVKSSDIKNS	C terminus amidation	
	LPASVVEDVKSSDIKNSKV	C terminus amidation	
	LPASVVEDVKSSDIKNSKVKRAPVN	C terminus amidation	
	NAQKTRMDNRYKREVD	N terminus acetylation; C terminus amidation	
	PLYTSEDE	N terminus acetylation	
	PTRNNYYTLSELLGAAQQEQNVPLYQRYVL		
	SNDPTREI		
	SPLYTSEDELGNDKT	N terminus acetylation; C terminus amidation	
Crustean cardioactive peptide (CCAP)	ANGYEGRDSIIDPK		$F_{2,9} = 5.380$, $P = 0.029$
	FAFLVIDTESIFLPKRANGYE	C terminus amidation	
	FAFLVIDTESIFLPKRANGYEGRDSIIDP	C terminus amidation	
	SMQGDND	Oxidation	
Diuretic hormone 31 (DH_{31})	APHNSRYMGYYGSNQDGQNPEYLLQTLARIRQAIIAEEDLENS		$F_{2,9} = 1.799$, $P = 0.22$
	GLDLGLGRGFSGSQAA		
	GLDLGLGRGFSGSQAAKH		
	GLDLGLGRGFSGSQAAKHLM	C terminus amidation	
	GLDLGLGRGFSGSQAAKHLMGLAAANFAGGP	Oxidation; C terminus amidation	
	GLDLGLGRGFSGSQAAKHLMGLAAANFAGGPG	C terminus amidation	
	LDLGLGRGFSGSQAAKHLMGLAAANFAGGP	N terminus acetylation; Oxidation; C terminus amidation	
Diuretic hormone 47 (DH_{47})	EENPLFGRENEPMDREAMGYILPKLMPRY	C terminus amidation	$F_{2,9} = 2.543$, $P = 0.13$
FMRFamide (FMRFa)	DKGHFLRF	C terminus amidation	$F_{2,9} = 13.002$, $P = 0.0022$
	GDLPANYEMEEGYDRPT	C terminus amidation	
	GNSDFLRF	C terminus amidation	
	NDNFMRF	C terminus amidation	
	PERNSNFLRF	C terminus amidation	
	STLYKNFARL	C terminus amidation	
	VLGDKSDQFIRF	C terminus amidation	
IDL-like (IDL)	AMAPHPLLLVSV	C terminus amidation	$F_{2,9} = 0.890$, $P = 0.44$
	IDLSRLYGHL		
	IDLSRLYGHLS		
	IDLSRLYGHLSS	C terminus amidation	
	IPHAVMAIDLSRLYGHL	C terminus amidation	
	IPHAVMAIDLSRLYGHLS		
	IPHAVMAIDLSRLYGHLSS		
	ISIQYLCDGAPDCSDGYDEDSRLCTAAKR	N terminus acetylation	
	LKPLGGVDKVAIALSESQTIED	N terminus acetylation	
Ion transport peptide (ITP)	SPAQRMSPLLSHHLS		
Myosuppressin (MYO)	AVAFIFVAMMASSNLSMASNLPLIYC	Oxidation	$F_{2,9} = 3.611$, $P = 0.071$
	DGLQKRQLCFALLERMDAPQEVSNDVMDNQLYERGI		
	FVAMMASSNLSMASNL	Oxidation	
	FVAMMASSNLSMASNLPLI	N terminus acetylation; Oxidation; C terminus amidation	
	LTVEDLVLVMNQCTVYAVAFIFVAMMASSNLSMAS	N terminus acetylation; Oxidation	
	QDVDHVFLRF	N terminus Pyroglutamination; C terminus amidation	
	RQLCFALLERMDAPQEVSNDV	N terminus acetylation; Oxidation	
	VLVMNQCTVYAVAFI	N terminus acetylation	
Myoinhibiting peptide (MIP)	AAIDVGSDPDIGIPKESDEMQM	Oxidation; C terminus amidation	$F_{2,9} = 1.652$, $P = 0.25$
	AAIDVGSDPDIGIPKESDEMQME	C terminus amidation	
	DPAWTNLKGIW	C terminus amidation	
	PEDEYAMKQLAT	N terminus acetylation	
	SAVLVIVGAIVCISMLPFSM	Oxidation; C terminus amidation	
	SEWGNFRGSW	C terminus amidation	
	VIVGAIVCISMLPFSMQAAIDVGSDPDIGIPKE	N terminus acetylation	
Natalisin (NTL)	CLGHCKFGGCVKRAARQDDMGP		$F_{2,9} = 6.866$, $P = 0.016$

Neuropeptide gene	Peptide	Possible modifications observed	Test of difference in neuropeptide abundances
		N terminus acetylation; Oxidation; C terminus amidation	
	DEPEEIDPFFTAR	C terminus amidation	
	YIVLDESPFMAAR	C terminus amidation	
Neuropeptide-like 1 (NPLP-1)			$F_{2,9} = 8.615$, $P = 0.0081$
	AGCLLLEAYGDSIAPE		
	AGYIRTLPDEDN	C terminus amidation	
	ANLAKNGQLPNYQNDA		
	ERDSGN		
	FLLQPAVDRILLQRVLMQPR	Oxidation	
	FLLQPAVDRILLQRVLMQPRN		
	FLLQPAVDRILLQRVLMQPRNH		
	GIESLARNGEL		
	GIESLARNGELH		
	GIESLARNGELHN		
	GIESLARNGELHNKREIEDLI	C terminus amidation	
	GIESLARNGELHNKREIEDLIDELY	C terminus amidation	
	GIESLARNGELHNKREIEDLIDELYE	C terminus amidation	
	GKRSIANLAKNGQLPNYQNDAEKRGIESLARNGELHN		
	HGPNDRSYDDMMKSDAERDSGNG	N terminus acetylation	
	KESYDDDYYRMAAF	C terminus amidation	
	LLLRASPAESIRGTSALWPDSAGCLLLE	N terminus acetylation; C terminus amidation	
	NIANLARSYSFPY	C terminus amidation	
	NLAALARAGYIRTLPDEDN	C terminus amidation	
	NLAALARAGYIRTLPDEDNG	C terminus amidation	
	NLAALARAGYIRTLPDEDNGKRSIANLAK	C terminus amidation	
	NLASIKAGYKQPF		
	NVAALLRQDKIHGPNDRSYDDMMKSDAERDSGNGD	C terminus amidation	
	NVASLARGGNLLY	C terminus amidation	
	NVASLARGGNLLYGKRNVAALLRQD	N terminus acetylation; C terminus amidation	
	SIANLAKNGQLPNYQND		
	SIANLAKNGQLPNYQNDA	C terminus amidation	
	SIANLAKNGQLPNYQNDAE	C terminus amidation	
	VDEMNKKKESYDDDYYRMAAF	N terminus acetylation; C terminus amidation	
	YDDMMKSDAERDSGNGD	N terminus acetylation	
	YIGSLARSGELNRF		
	YIGSLARSGELNRFHND	C terminus amidation	
Pheromone biosynthesis activating neuropeptide (PBAN)			$F_{2,9} = 5.377$, $P = 0.029$
	AQLENYDKAITIYQDVAMSSLESSLLKYSAKE		
	HNKMNFTPRL	C terminus amidation	
	KMSALWFGPRL	C terminus amidation	
	NPSSDELLKNTNLDREQLVALLEMLQESPWAVVALNE	C terminus amidation	
	TMAAKHHQSIAEMYES	N terminus acetylation; Oxidation; C terminus amidation	
RYamide (RYa)			$F_{2,9} = 4.033$, $P = 0.056$
	ADKAAKTAGKHVIVAPR		
	ADKAAKTAGKHVIVAPRNDKFFLASRY	C terminus amidation	
	ALTNRSGYN		
	ANDRPFMMGMRY	C terminus amidation	
	ASRYGKRSGGEMISNAAQAALVFPVPP	Oxidation	
	DAMKPSELQDHLRRCHP	N terminus acetylation; Oxidation; C terminus amidation	
	GGAAHQAVQLITRGMANSDTTESEDTGIRRCW	Oxidation; C terminus amidation	
	LNAVLEFYIGTVEA	C terminus amidation	
	MKPSEL	C terminus amidation	
	MMTDAMSESKKKCRQY	N terminus acetylation; Oxidation	
	NDKFFLASRY	C terminus amidation	
Tachykinin (TK)			$F_{2,9} = 5.882$, $P = 0.023$
	APNGFFGMR	Oxidation; C terminus amidation	
	DLETVLLPEES		
	ESKRAPNGFFGMR	C terminus amidation	
	PSGFTGVRGKKSFEDEDFEMR	N terminus acetylation; Oxidation	
	PSRSAGFFGMR	C terminus amidation	
	SFEDEDFEMRDIED		
	YPYEFRGKFVGV		
	YPYEFRGKFVGVR	C terminus amidation	
Short neuropeptide F (sNPF)			$F_{2,9} = 2.074$, $P = 0.18$
	LRFGRRSDPSLIQASPYMLSAAQDAAE		
	SDPSLIQASPYMLSAAQDAAEIAN	C terminus amidation	
SIFamide (SIFa)			$F_{2,9} = 0.297$, $P = 0.75$
	SAMCEI	N terminus acetylation; Oxidation; C terminus Amidation	
	TYRKPPFNGSIF	C terminus amidation	
Sulfakinin (SK)			$F_{2,9} = 8.756$, $P = 0.0077$
	EDFDDYGHLRY	C terminus amidation	
	GPAGASVPTEANRRI		
	MKLLLVAMCLILMACNDGASAGPAGASVPTEANRRIRS	Oxidation	
	QNSDDYGHLRF	N terminus Pyroglutamination; C terminus Amidation	
	QTYFMMKLLLVAMCLILMACNDGASAGPAGASVPTEANRRIRS	Oxidation	

Also presented are the results of ANOVA for differences in the abundance of that neuropeptide in different behavioural states.
